# Emerging trends in genome integration tools for precision engineering of diverse bacterial species

**DOI:** 10.1093/synbio/ysaf019

**Published:** 2025-12-11

**Authors:** Riesa K W Rohmat, Thea C T Irvine, Shivang Hina-Nilesh Joshi, Andrew M Bailey, Christopher Jenkins, David Ulaeto, Pierre Buscaill, Thomas E Gorochowski

**Affiliations:** School of Biological Sciences, University of Bristol, Bristol BS8 1TQ, United Kingdom; School of Biological Sciences, University of Bristol, Bristol BS8 1TQ, United Kingdom; School of Biological Sciences, University of Bristol, Bristol BS8 1TQ, United Kingdom; School of Biological Sciences, University of Bristol, Bristol BS8 1TQ, United Kingdom; Defence Science and Technology Laboratory, Porton Down, Wiltshire SP4 0JQ, United Kingdom; Defence Science and Technology Laboratory, Porton Down, Wiltshire SP4 0JQ, United Kingdom; School of Biological Sciences, University of Bristol, Bristol BS8 1TQ, United Kingdom; School of Biological Sciences, University of Bristol, Bristol BS8 1TQ, United Kingdom

**Keywords:** genome integration, genome editing, synthetic genomics, bacteria, synthetic biology

## Abstract

The ability to precisely insert DNA payloads into a genome enables the comprehensive engineering of cellular phenotypes and the creation of new biotechnologies. To achieve such modifications, the most widely used techniques rely on a host cell’s native DNA repair mechanisms like homologous recombination, which hampers their broader use in organisms lacking these capabilities. Here, we explore the current landscape of genome integration systems with a particular focus on those that function in bacteria and are precise, self-contained, and portable, placing minimal requirements on the host cell. Through a historical analysis, we observe long-term use of recombineering technologies, a recent rise in the use of CRISPR-guided systems that consist of associated integrase machinery, and growing efforts to modify non-model organisms. Looking forward, we highlight some of the remaining challenges and how synthetic genomics may offer a way to create bacterial strains optimized for extensive long-term modification. As the field of synthetic biology sets its sights on real-world impact, the effective engineering of genomes will be critical to shaping the robust phenotypes that applications demand.

## Graphical Abstract

**Graphical Abstract:**
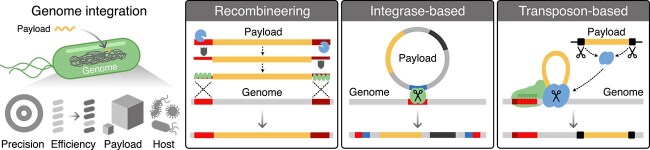

## Introduction

The field of synthetic biology has rapidly progressed in recent years, transforming our ability to engineer biological systems for applications ranging from sustainable bio-based production to new forms of medicine [1]. Central to this revolution has been the capacity to precisely manipulate genetic material, enabling the implementation of novel functionalities within living cells. While early approaches to synthetic biology relied heavily on plasmid-based systems for gene delivery and expression, these systems have inherent instability [2], can impose a substantial metabolic burden on the host [3], and are often impractical for deployment of engineered systems into the real-world. This has prompted the field to shift towards genome engineering to enable the development of more reliable, stable, and industrially viable bioengineered products [4–6].

This transition has been supported by genome integration technologies that support the precise and targeted insertion of DNA payloads directly into a host cell’s chromosome. Unlike traditional cloning approaches that rely on restriction enzymes and are typically performed *in vitro*, modern genome integration methods harness diverse molecular mechanisms used in nature for horizontal gene transfer [7], site-specific recombination [8], and DNA repair *in vivo* [9]. These tools open opportunities for metabolic engineering, pathway optimization, and the creation of robust microbial cell factories capable of sustained production under industrial conditions. The ability to achieve stable and heritable modifications without the complications associated with plasmid maintenance has been particularly valuable for applications requiring long-term genetic stability [10] and consistent expression in highly variable environments (e.g. complex soil communities [11]).

Historically, most genome integration tools have exploited the host cell’s native ability to perform homologous recombination. While this remains a powerful method that is still widely used today, homologous recombination is not always efficient across organisms, limiting its use in diverse species. To overcome this issue, three broadly applicable approaches for bacterial genome integration have emerged: (i) recombineering, (ii) phage integrase mediated integration, and (iii) transposon-based integration. Recombineering is the simplest and longest used strategy for genome integration. It relies on the hosts’ native DNA repair mechanism to incorporate a payload sequence flanked by homology arms that direct the insertion. This method is enhanced by exogenously provided proteins from the phage recombination machinery [9], which accelerates the recombination process and significantly improves integration efficiency even with relatively short homology regions. Phage integrase-based methods exploit site-specific recombinases that mediate integration at predetermined attachment sites, offering higher efficiency and specificity, as well as the capacity to handle larger DNA payloads [12–14]. Transposable elements are yet another class with diverse mechanisms and features for genome integration. These include systems capable of random insertions (Tn5, Mu) [15] or site specificity (Tn7) [16], as well as newly discovered programmable insertions via CRISPR-associated transposons (CAST) [6, 17].

Despite the development of numerous genome integration tools, several challenges continue to limit their broader adoption. Most existing tools are hampered by host-specificity, as tools evolved for a particular organism often function poorly when used in others that are distantly related [18, 19]. In addition, integration bias [20], off-target effects [17, 21], and the accumulation of genomic scars from repeated modifications [22, 23], all cause issues when modifying genomes. There is also an increasing demand for tools able to simultaneously make several modifications to a host cell’s chromosome, driving the development of entirely new classes of genome engineering tools that extend beyond those found in nature [24, 25].

Beyond the specific features of the genome integration tools themselves, other bottlenecks impact their practical use. Perhaps most significantly, building genome-integrated systems takes more time than plasmids-based ones, due to the many steps involved to construct, clean-up, and verify each genomic modification. Furthermore, genome-integrated systems function at much lower expression levels due to their near single-copy number, and can be affected by their genomic context, i.e. the local sequence composition, structural chromosome conformation, and transcriptional activity around their point of insertion in the genome. As a result, multiple rounds of time-consuming optimization are often required before a functional system is achieved. This challenge is further compounded by the relatively little knowledge about how genome integrated systems should be built [26], in contrast to the vast literature on their plasmid-based counterparts [27].

In this review, we aim to provide an overview of the current landscape of targeted genome integration technologies for bacteria, with a particular focus on methods that are precise, self-contained, and portable across diverse bacterial species. We demonstrate the rapid evolution of this field and emerging trends, as well as identifying growing interest in the genome engineering of non-model organisms. We also highlight key technical challenges that continue to hamper efforts. Finally, we explore the new technologies being developed and discuss the roles they will likely play in enabling more comprehensive modification of natural and synthetic genomes. As synthetic biology moves beyond the proof-of-concept stage, the creation of robust, efficient, and broadly applicable genome integration tools will be critical for building reliable biotechnologies with dependable functions and minimized risk of unforeseen effects.

## Results

**Figure 1:**
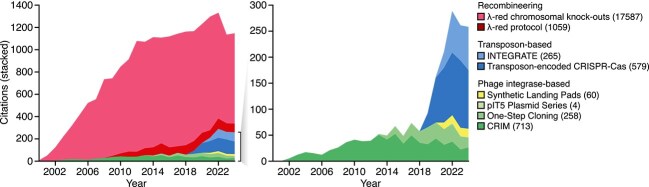
Citations of key bacterial genome integration papers over time. Citation counts per year were calculated using Google Scholar. The following references were used for each method: Lambda Red chromosomal knock-outs [9]; Lambda Red protocol [28]; INTEGRATE [17]; Transposon-encoded CRISPR-Cas [6]; Synthetic Landing Pads [4]; pIT5 Plasmid Series [53]; One-Step Cloning [22]; CRIM [8]. Right plot shows zoomed in data for only transposon-based and phage integrase-based methods. Total number of citations until the end of 2024 are shown in parenthesis. Further details about each method can be found in Fig. 4.

The past few decades have seen major advances in our ability to modify genomes through the discovery of diverse biochemical processes and molecular tools able to locate, cut, and ligate DNA. To better assess the emergence and usage of bacterial genome integration tools over time, we extracted the numbers of citations for key papers covering the major targeted integration methods in use today (i.e. recombineering [9, 28], phage integrase-mediated integration [22], and CRISPR-targeted transposon-based integration [6, 17]). We found a rapid uptake of new methods, with distinct technological waves shaping the field’s development (Fig. 1). In the year 2000, recombineering emerged as an early method. In contrast, phage integrase-based approaches have seen consistent, low-level usage over two decades, while recent years have witnessed a steep rise in the application of CRISPR-targeted transposon systems. A deeper analysis of use cases revealed clear trends in efforts to simplify integration protocols and widen their use for modification of diverse bacterial species [14, 29, 30]. In the following sections, we examine these methods further, comparing their benefits and limitations, and discussing the field’s broader progression towards increasingly versatile tools for genome engineering.

### Recombineering

Recombineering is the most widely adopted method for bacterial genome modification, in part due to its use of the native DNA repair machinery. However, for broader practical applications and higher efficiency, exogenously provided recombination machinery is needed. Some bacteria, such as *Bacillus subtilis* and various cyanobacteria species, readily incorporate heterologous DNA into their chromosomes, provided the payload is flanked by homologous arms [31, 32]. However, this natural ability is rare and often absent in bacteria of interest for biotechnology. To address this, the widely used lambda Red method (Fig. 1a), initially developed in *Escherichia coli*, employs bacteriophage proteins (Red$\alpha $/$\beta $/$\gamma $) to significantly improve efficiency over the native RecA-dependent homologous recombination pathway while maintaining high precision using only $\sim $50 bp of homology [9]. This requirement of shorter homology arms makes lambda Red much easier to use as homology sequences can be easily incorporated into primer overhangs. This enables simple customization of insertion sites for a range of modifications covering gene knockouts, knock-ins, substitutions, and even point mutations.

Two major types of recombineering system are typically used for bacterial genome engineering: the lambda Red system (derived from bacteriophage lambda) [28] and the RecET system (derived from the Rac prophage) [33]. The lambda Red system is widely used due to its high efficiency, ease of implementation, and broad compatibility with many *E. coli* strains. However, the RecET system can offer advantages in specific genetic contexts or strains where lambda Red shows reduced effectiveness [34]. These systems also differ in their substrate preferences: the RecET system has higher efficiency for linear-plus-linear homologous recombination, while lambda Red favours linear-plus-circular homologous recombination [35].

The lambda Red system operates using three phage-encoded proteins with distinct but coordinated functions: Exo degrades one strand of double-stranded DNA (dsDNA) to generate single-stranded DNA templates, Beta promotes strand pairing between the processed DNA and homologous regions in the target sequence, and Gam acts as an inhibitor of the *E. coli* RecBCD nuclease complex that would otherwise degrade linear DNA substrates [9, 28, 36–38]. This nuclease inhibition is crucial for the system to function effectively. Insufficient blocking of RecBCD significantly reduces recombineering efficiency, particularly in bacterial species outside *E. coli* where *recBC* mutant strains may be required. The RecET system employs RecE and RecT proteins that perform analogous functions to Exo and Beta but the method lacks an endogenous nuclease inhibitor. Interestingly, Gam can enhance RecET system performance despite being naturally exclusive to the Lambda phage system [35].

**Figure 2:**
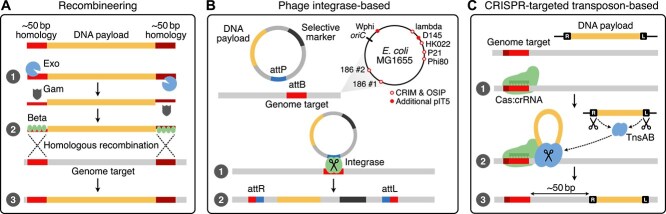
Common targeted genome integration systems used in bacteria. (a) The lambda Red recombineering system allows for integration using $\sim $50 bp homologous arms at both ends of the DNA payload and employs the proteins Exo, Beta, and Gam to facilitate homologous recombination (HR) into the host chromosome. Exo processes dsDNA by removing one strand to create a single-stranded template, while Beta aligns the recombineering substrate with the chromosomal sequence, and Gam (not shown) protects the dsDNA from degradation. (b) Phage-based genome integration promotes recombination between the bacterial DNA sequence (the bacterial attachment site, attB) and the phage DNA sequence (the phage attachment site, attP). The designated phage attachment sites depend on the plasmid system used. The original CRIM system had five sites (lambda, HK022, Phi80, P21, and P22, not shown), while OSIP added two sites (186#1 and 186#2) without P22, and the pIT5 plasmid added Wphi and D145, resulting in a total of eight sites. (c) CRISPR-targeted transposon-based genome integration methods use a gRNA (crRNA) to target a specific genomic sequence at the PAM for recognition by the TniQ-Cascade complex, which recruits the transposition proteins TnsA, TnsB, and TnsC. TnsC bridges the Cascade complex to the transposase machinery, positioning the integration site, while TnsA cleaves donor DNA and TnsB facilitates its insertion into the genome.

Genome integration via recombineering follows a well-defined three-stage process (Fig. 2a) [9, 28]. First, cells are transformed with linear dsDNA containing homology regions matching the target insertion site. Second, the Exo protein processes this DNA to generate single-stranded templates, which are then bound and protected by Beta proteins. Gam provides additional protection by inhibiting host nucleases that would otherwise degrade the DNA payload. Finally, the Beta-ssDNA complex, working in conjunction with the host’s native RecA protein, locates the homologous target sequence and enables strand invasion and the formation of a Holliday junction—a cruciform DNA structure where two dsDNA molecules are joined together through reciprocal strand exchange. Typically, selection markers are included in the payload to simplify identification of successful recombinants. An issue with this approach is that sequential modifications require different selection markers at each cycle. Therefore, approaches that flank selection markers with recombination sites have been developed using the cognate recombinase enzyme to excise the marker once successful integration has been verified [39, 40]. Recent advances have also incorporated CRISPR endonucleases to cleave unmodified target sequences, ensuring that only recombinants with successful integration survive the CRISPR-mediated genome targeting [41], removing the need for selective markers completely.

While recombineering is a powerful genome engineering approach, its successful application depends on specific experimental conditions and strain compatibility, which can complicate experimental design. In addition, prior knowledge of the target sequence is essential, limiting its use in poorly characterized organisms [41]. Recombineering is also less reliable when targeting repetitive DNA sequences, as recombination errors are more likely to occur. Another limitation is host cell tolerance, as prolonged expression of phage-derived proteins is often toxic, reducing bacterial viability. A further constraint in the efficiency of this method is sensitivity to the size of the DNA payload. Although integration of fragments up to $\sim $50 kb have been reported [38], efficiency decreases substantially with increasing fragment size, with optimal performance typically seen for 3–4 kb inserts [28]. As a result, larger DNA payloads must be divided into smaller segments and sequentially integrated, which extends the experimental timeline. This size limitation likely arises from the short homology arms (30–50 bp) that are used, which may lack sufficient specificity and stability needed for efficient recombination of larger payloads.

Despite these technical challenges, recombineering remains one of the most widely used methods for integrating relatively small DNA payloads into bacterial genomes. It offers several compelling advantages. For example, it is precise allowing for the targeting of any sequence using only short homology sequences and can be used to edit various DNA substrates, including chromosomes [28, 42]. Importantly, recombineering facilitates seamless modifications that avoid the introduction of undesired genetic scars [28], making it particularly valuable for applications that require ‘clean’ genomic edits. These features have established recombineering as a foundational tool in bacterial genome engineering, particularly for targeted modifications in well-characterized model organisms.

### Phage integrase-mediated integration

Bacteriophages have evolved sophisticated site-specific recombination systems that enable the insertion of their DNA at predetermined sites within the genomes of their bacterial host [43, 44]. These natural integration mechanisms have been harnessed for genome engineering applications, providing precise and efficient methods for targeted DNA insertion [44]. Phage integrases are among the longest-established tools for targeted payload delivery into bacterial genomes and their adoption has accelerated with the development of dedicated plasmids to simplify their use.

The site-specific recombinases, employed by bacteriophages, belong to two evolutionarily distinct families: tyrosine recombinases (e.g. phage HK022) and serine recombinases (e.g. phage $\varphi $C31) [43]. Both families can integrate large DNA payloads, with some systems capable of handling >100 kb of genetic material [45]. These phage-derived integrases mediate recombination between their respective target DNA sequences, referred to as attachment sites. The bacterial attachment site (attB) is located in the host genome and phage attachment site (attP) is on the incoming exogenous DNA (Fig. 2b). The integrase binds these sites typically forming a multimeric complex that then catalyses DNA cleavage, strand exchange and ligation. The result of this process is the integration of the entire plasmid flanked by modified attachment sites, designated as ‘left’ (attL) and ‘right’ (attR). The genetic payload remains stably integrated, unless the specific excisionase (Xis) proteins are present to mediate the reversal of the integration. [46].

**Figure 3:**
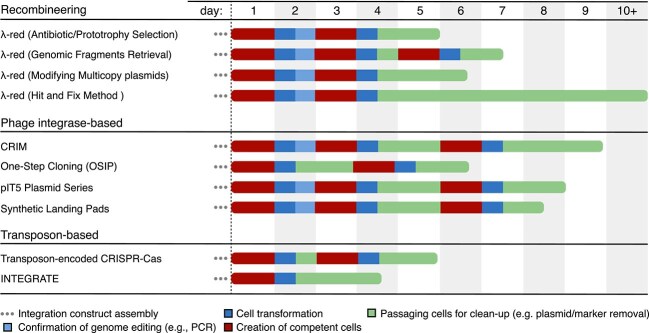
Timelines for different bacterial genome integration methods. The coloured bars represent the steps and the length of time typically taken to complete. The three dots represent plasmid assembly required for strain modification and integration, with variable timescales depending on the use case. Except for the Synthetic Landing Pads method [4], all plasmids are publicly available (e.g. from Addgene). Each approach starts with the production of competent target cells. Some methods require the pre-expression of integration-related genes (e.g. lambda Red method: introduction of Lambda Red genes [28]; CRIM method: introduction of integrase gene [8]). In contrast, OSIP and transposon-based approaches can skip the gene expression steps and go straight to the payload integration stage. Recombineering is typically performed in four different ways (top–bottom in section): (i) drug resistance or prototrophic marker inserted into bacterial chromosome and insertion confirmed using selection and PCR [28]; (ii) genomic retrieval via recombineering to extract segment DNA from bacterial chromosome, which requires sub-cloning to other bacterial strain for confirmation (sub-cloning method) up to 3 days [163]; (iii) modify multi-copy plasmid; confirmation required PCR and restriction analysis to confirm alterations while avoiding unwanted plasmid multimer formation up to 3 days [28]; and (iv) ‘Hit and Fix’ method using two-step recombineering procedure in which a heterologous ‘Hit’ sequence is introduced and detected using colony hybridization. In the second ‘Fix’ phase, the original sequence is restored except for the intended mutation, ensuring precise genetic change while removing undesirable markers (up to 2 weeks) [164].

The Conditional-Replication, Integration, and Modular (CRIM) plasmid system was one of the first systematic approaches to harness phage integrases for genome engineering [8]. CRIM consists of a comprehensive set of plasmids designed to support the insertion and removal of genetic payloads at natural integrase attachment sites within the *E. coli* genome. CRIM-based genome integration involves two sequential steps. First, target cells are transformed with a helper plasmid expressing the appropriate phage integrase. Next, these cells are further transformed with a non-replicative payload plasmid (R6K$\gamma $ origin of replication) carrying the attP site corresponding to the phage being expressed [8]. The CRIM system also has a set of additional plasmids with Int/Xis genes to remove the genetic payload if required. The CRIM system can make use of five distinct integration sites (P22, Lambda, HK022, P21, and Phi80) and the entire process typically requires between 5–8 days (Fig. 3) [8]. While effective, the multi-step nature of CRIM and its long protocol ultimately led to the development of quicker and more streamlined methods.

**Figure 4:**
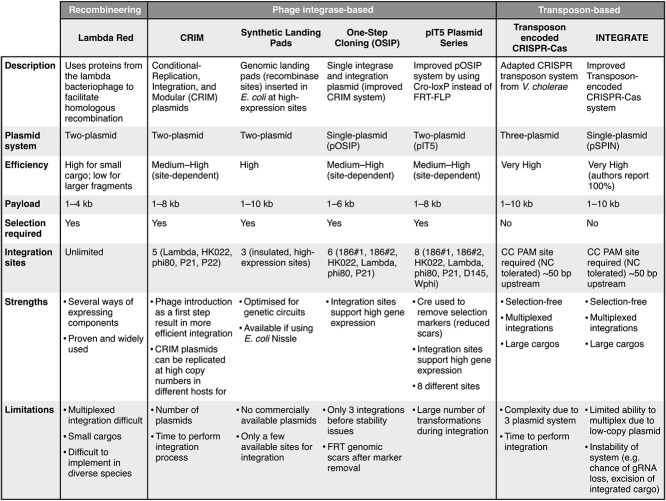
Key features of widely used precise and targeted bacterial genome integration systems. Reported efficiencies are based on author claims [4, 6, 8, 17, 22, 28, 53]. For payload size, we state the typical range of lengths used with the largest successfully reported integration in parentheses. Most routine lambda Red recombineering uses PCR products of $\sim $1 kb. Efficiencies are found to drop significantly for inserts larger than 4 kb in length. Typical CRIM plasmids used in published studies are 5–8 kb in size. Plasmid count for each system excludes excision plasmids used by some of the methods.

The One-Step Integration Plasmid (OSIP) system, also known as Clonetegration, emerged in 2013 as a significant improvement on CRIM [22]. OSIP consolidates integration and integrase expression into a single vector, with the integrase protein expression controlled by a temperature-sensitive lambda-CI repressor gene. OSIP expanded the available integration sites to six (186#1, 186#2, Lambda, HK022, Phi80, and P22), offering users greater flexibility in site selection. A key innovation was the use of the Flp recombination system [47] for marker removal. In this case, markers are flanked by Flp recombination target (FRT) sites and the regions excised through the expression of the Flp recombinase. Unfortunately, this approach introduces genomic FRT scars after marker removal that can limit the number of integration rounds before cellular toxicity and unwanted recombination events become problematic. In our experience, there are noticeable growth defects once 5–6 FRT scars are present. Despite this limitation, OSIP represents a notable improvement in the efficiency of integrase-mediated integration (Figs 3 and 4). Similarly, a modular cloning compatible CRIM system, CRIMoClo [48], has been developed with the intention of enabling cloning compatibility with the growing list of synthetic biology toolkits [49–52]. However, the issue of limited numbers of stable genome modifications due to the repeated use of FRT sequences remains.

More recently, the pIT5 plasmid series has been developed to address the scar accumulation problem while also adding two new integration sites (Wphi and D145) within the *E. coli* genome [53]. This system uses a two-plasmid strategy that pre-expresses a specific integrase in the target cells, similar to the original CRIM system. This has been shown to enable higher integration efficiency than the original single-plasmid OSIP method. The key improvement of pIT5 over OSIP was the replacement of the Flp-FRT recombination system for selection marker removal with a Cre-loxP recombination system [39, 40], in which the Cre recombinase is delivered using a cosmid. This modification enables the removal of markers while leaving inactive loxP scars that no longer interact with Cre. This prevents large-scale chromosomal rearrangements and cell death. Integration of a payload using pIT5 can be completed in 5 days, representing a further improvement compared with the original CRIM workflow (Figs 3 and 4).

Beyond exploiting naturally occurring attB sites within a genome, researchers have more recently developed ‘landing pads’ containing synthetic attachment sites (i.e. attachment sites not native to the host genome) that can be strategically positioned throughout a genome. This approach has been successfully applied the integration of large genetic circuits [4], including application in clinically relevant strains like *E. coli* Nissle 1917 [54]. In this case, insulated landing pads flanked by strong bidirectional transcriptional terminators were designed to minimize transcriptional interference between the integrated payload and the genome. Insertion sites of these landing pads were also selected based on RNA sequencing data to identify genomic loci supporting high-levels of gene expression [4]. While the use of landing pads offers advantages in expression control and genomic positioning, several prerequisites are required for their successful use. For example, whole genome sequences and transcriptomics data are required, and a set of integrases must be available for the host of interest. These requirements can limit genome engineering in non-model bacteria, which often lack such resources. Nevertheless, novel integrases continue to be discovered in both academic [55, 56] and industrial [57] contexts, expanding the repertoire of tools that could be used for engineering the genome of diverse organisms.

In summary, phage integrases offer several compelling advantages for genome engineering applications. Their high target-site specificity minimizes off-target effects, and they are capable of integrating large DNA payloads [22, 48, 53], offering advantages over homologous recombination-based strategies. Multi-site integration using orthogonal integrases simultaneously further enhance their utility, although integration efficiencies vary across different integrase-site combinations [4, 22, 48]. Careful selection and optimization of the system is therefore essential to fully leverage the potential of phage integrase-mediated integration.

### CRISPR-targeted transposon-based integration

Transposons, often referred to as ‘jumping genes’, are mobile genetic element capable of moving between genomic locations [58]. This mobility allows them to integrate into new DNA regions, generating genetic diversity within populations that is often exploited for adaptation [59–64]. A well-known consequence of transposon-activity is the horizontal transfer of antibiotic resistance genes within and across bacterial species. In these cases, transposons facilitate the spread of resistance by mediating gene transfer via plasmids and other mobile genetic elements [65, 66].

Transposable elements are broadly classified into two major categories based on their mobilization mechanisms: Class I (retrotransposons) and Class II (DNA transposons) [67]. Class I retrotransposons operate via an RNA intermediate, which is then reverse transcribed into a cDNA copy before reintegration into the genome [67]. In contrast, Class II DNA transposons use a ‘cut-and-paste’ mechanism, where a transposase enzyme excises the DNA element, which is then inserted into a new genomic site without replication [67]. DNA transposons typically contain long terminal repeats that flank the payload DNA [68] and are recognized by the transposase for excision and integration [42, 66]. These mechanistic differences have important implications for genome engineering applications. Retrotransposons increase copy number with each mobilization event, leading to genome expansion, whereas DNA transposons maintain a constant copy number, offering a more stable integration outcome. Although Class I retrotransposons are predominantly found in eukaryotes, they have also evolved in prokaryotes [42, 66]. However, their tendency to create unstable genomic modifications limits their utility for engineering applications. As a result, Class II DNA transposons are preferred for bacterial genome integration and are our focus here.

Tn5 is a homodimeric transposase (Tnp) that is widely used for generating genomic libraries through transposon mutagenesis [15, 69, 70]. With no dedicated regulatory proteins for site recognition, Tn5 transposons, and others like it, are often described as performing random insertions. However, they do in fact exhibit distinct biases towards specific genomic features. These preferences include AT- and GC-rich regions [71, 72], euchromatic regions with particular chromatin characteristics [73, 74], and distinct patterns between intergenic and intragenic sequences [75]. Integration preferences vary substantially depending on the host organism’s genomic landscape and the specific transposon system being used [76]. In contrast, the well-characterized Tn7 transposon from *E. coli*, which also belongs to the Class II DNA transposon family, enables targeted insertion of DNA payloads [77, 78]. Tn7 utilizes a heteromeric transposase complex comprised of TnsA and TnsB proteins, with additional proteins TnsC, TnsD, and TnsE that coordinate recognition of the attachment site attTn7, located downstream of the *glmS* gene in *E. coli*, or by recognizing mobile plasmids, to facilitate horizontal gene transfer [16, 79–81]. Like Tn5, Tn7 plays a key role in bacterial genome modification and is widely used in biotechnological applications to facilitate stable gene insertion [82–85].

Unfortunately, both Tn5 and Tn7 place limitations on where the integration of a DNA payload can be targeted. Tn5 is highly random with little control over insertion sites, while Tn7 is highly specific and limited by the availability of innate attachment sites in the host genome [82, 85, 86]. In recent years, comparative genomics has uncovered diverse CAST systems that combine Cas-mediated RNA-guided DNA targeting with the key advantage of transposons—integration without the need for double stranded breaks or host homologous recombination pathways [78, 87–91]. This combination of features enables user-directed, site-specific insertion, overcoming the limitations of both classical transposons and traditional CRISPR-Cas editing. While precise DNA integration can be achieved following Cas9- or Cas12-induced cleavage, such approaches are often inefficient, relying on double-stranded breaks, and their efficiency varies substantially depending on cell type [92]. In essence, the discovery of these CRISPR-associated transposons, with Tn7-like multi-protein structures, has revolutionized targeted transposition capabilities [91].

Tn7-like CAST systems, which lack interference nucleases, have most likely been evolutionary repurposed for transposon mobility rather than host immunity [6, 86, 93]. The crRNAs employed by these CAST systems differ significantly from those found in typical CRISPR-Cas immune systems. For example, type V-K CASTs use short, atypical crRNAs to target tRNA genes, while type I-B systems rely on dual selector proteins such as TniQ for crRNA-guided integration and TnsD for targeting the DNA protospacer to guide insertion [88, 89]. Similarly, type I-F CASTs utilize non-canonical crRNAs with variable repeat structures to recognize target sites, illustrating diverse evolutionary solutions that converge on RNA-guided DNA transposition [89].

V-K CASTs subtypes of particular interest include the shCAST subtype found in the cyanobacteria *Scytonema hofmanni* and the acCAST subtype found in the cyanobacteria *Anabaena cylindrica* [92]. Both shCAST and acCAST couple a minimal Cas12k system with Tn7-like transposase genes (*tnsA*, *tnsB*, *tnsC*, *tniQ*) and crRNA for site specific unidirectional integration. The two systems share similar protospacer adjacent motif (PAM) sequences of the form ‘NGTN’ with insertions occurring 60–66 bp (shCAST) and 49–56 bp (acCAST) downstream of the protospacer, respectively [92]. Furthermore, shCAST has been demonstrated to have an 80% insertion efficiency in *E. coli* without selection and is able to tolerate a broad range of donor DNA lengths (0.5–10 kb), with slightly higher efficiencies seen for smaller payloads [92]. Despite the functional similarities, the spatial offset in the point of integration distinguishes acCAST from shCAST and may reflect system-specific structural differences in DNA-targeting or transposase complex positioning. For example, the 10–11 bp difference in integration position seen in acCAST compared with shCAST likely stems from a one-turn shift in DNA helix positioning [92].

Another CAST system that has received significant and growing interest is the Tn6677 Type I-F Tn7-like CAST from *Vibrio cholerae* referred to as vcCAST. This system is comprised of Cas6, Cas7, Cas8–5 fusion, and TniQ [6, 78, 94] integrated within its CRISPR operon. These proteins form a Cascade–TniQ co-complex that guides transposition to target sites and has an optimal ‘CC’ PAM (although ‘CN’ PAMs are also tolerated) [6, 95]. Like all CRISPR-Cas systems, vcCAST relies on specific PAM sequences, which restricts the range of compatible target sites [6]. However, because vcCAST requires a simple PAM motif, this limitation is relatively minor in practice. The RNA-guided complex scans the genome, binds to target sequences through complementary base pairing, and recruits the transposition machinery consisting of proteins TnsA, TnsB, and TnsC. TnsA and TnsB, form the transposase complex responsible for cutting and translocating the DNA payload, while TnsC acts as a molecular bridge linking the cascade complex to the transposase machinery [81, 94, 96]. This coordination positions the integration site 47–51 bp downstream of the target DNA, varying depending on the protospacer and features of the surrounding DNA sequence [6, 88, 96]. Due to these structural constraints of the transposition complex, integration distances are variable, making this system unsuitable for generating precise in-frame mutations. Unlike the V-K systems mentioned earlier, which are exclusively unidirectional, orientation can vary when using the vcCAST system. For example, one crRNA yielded a 39:1 insertion ratio in one direction and another yielded a 1:1 insertion ratio [6], suggesting additional determinants that regulate orientation (e.g. the sequence or structural context).

The most prominent Tn6677 vcCAST system to date forms part of the INTEGRATE system, which enables precise multiplexed integration of DNA payloads in diverse bacterial species including *Klebsiella oxytoca* and *Pseudomonas putida* (Fig. 2c) [6]. This system was a significant advancement in transposon-based integration. Its high integration efficiency (which can reach near to 100%) means that the use of antibiotic selection markers is not always needed, as small scale screening (e.g. via colony PCR) allows for correctly edited clones to be efficiently found. Optimal integration efficiencies with vcCAST Tn667 expressed from a three plasmid system were reportedly observed for payload sizes of 775 bp, with reduced efficiency for both longer and shorter payloads [6]. Yet, the vcCAST system expressed from a single-plasmid called pSPIN demonstrates impressive payload capacity being able to integrate DNA fragments up to $\sim $10 kb [94]. This system has also been shown able to support multi-spacer arrays, simultaneously performing insertions at multiple chromosomal sites, with few unwanted off-target effects [94, 97], and the integration process is rapid, typically requiring less than one week from preparation to completion when constructs are readily available (Fig. 3 and Fig. 4) [21]. The main limitations of the INTEGRATE system are that current multiplexing capabilities only allow for the delivery of identical payloads at different genomic sites, and the pSPIN plasmid replicates via the low-copy pSC101 origin of replication, inherently restricting the number of simultaneous integration events achievable in a single application. However, its incorporation of a rep101 temperature-sensitive origin of replication simplifies plasmid curing and streamlines iterative rounds of genomic integrations [21, 94].

The reliance of CRISPR-associated transposons on flanking terminal inverted repeats (TIRs) for payload recognition and mobilization results in these TIR sequences being incorporated into the genome upon integration [6, 21, 92]. Furthermore, the joining events between the target DNA and payload, mediated by proteins TnsA and TnsB, are staggered by 5 bp, leading to small gaps that are repaired by host DNA repair processes. This repair results in a 5 bp target site duplication flanking the inserted sequence [98]. Consequently, these systems cannot be considered scar-free. Incorporation of the TIR elements may have unexpected consequences. For example, a cryptic promoter has been identified within the right transposon end of the vcCAST system, potentially leading to unintended transcriptional activation [6, 97]. To overcome this, it has been shown that only around 105 bp of the left TIR and 47 bp of the right TIR are necessary for efficient RNA-guided DNA integration, which corresponds to three and two intact putative TnsB-binding sites, respectively [6]. The INTEGRATE mini-Tn tools, to which pSPIN belongs, therefore exploit a truncated 57 bp right transposon end to circumvent cryptic promoter activity [94]. Because the pSPIN system does not require selection markers, this further reduces the overall payload size, eliminating the need for additional elements to be included. In contrast, the absence of such selective markers complicates the identification of successful integration events, often necessitating laborious colony screening. Perhaps the most significant limitation of the pSPIN system is its constitutively active CAST operon, where the guide protospacer can potentially be incorrectly recognized as a genomic target within the plasmid itself [21, 94]. Such self-insertion events reduce overall integration efficiency, by depleting the pool of functional plasmids and available payload [21, 94]. This effect is particularly problematic at challenging genomic loci, such as sites with low accessibility near the replication terminus. To address this, later iterations of the pSPIN tool relocated the CRISPR array upstream of the mini-Tn element, thereby minimizing disruption of the Cascade-transposon operon in the event of self-insertion [21]. However, due to the excision-based mobilization of DNA payload this strategy does not resolve the problem of payload depletion from the plasmid pool.

Despite these limitations, CRISPR-targeted transposon systems represent a powerful addition to the genome engineering toolkit, offering unique capabilities for rapid, and multiplexed DNA integration that complement existing approaches such as recombineering and phage integrase-mediated insertion. These systems not only deepen our understanding of how CRISPR-Cas components can be retooled by transposons but also offer versatile tools for biotechnology. Recent developments have expanded the transposon-based toolkit with the development of bridge RNAs, which utilize insertion sequence (IS110) elements expressing structured non-coding RNAs [99]. These bridge RNAs bind both target and donor DNA via modular binding loops, enabling sequence-specific recombination mediated by a single recombinase enzyme [99]. Such discoveries highlight the rapidly evolving field of transposon-based genome engineering and reveal that novel mechanisms continue to drive innovations in precise genome modification.

### Genome integration technologies for non-model microbes

Many of the methods described so far, such as phage-based integration and transposon-based integration, have been successfully applied in diverse bacteria. For example, the OSIP system works effectively in *Salmonella typhimurium* [22] and INTEGRATE has been used successfully in *K. oxytoca* and *Pseudomonas putida* [17, 21]. More broadly, a type I-F CAST system called *Pse*CAST has enabled double-stranded break-free DNA integration in human cells, although at low efficiency [100], while other CAST systems have been used as part of a dual-plasmid strategy to achieved near-100% targeted gene disruption and large >11 kb integrations in *Burkholderia thailandensis*, *P. putida*, and *Agrobacterium fabrum* [101]. By exploiting efficient DNA repair pathways in some bacteria, a thermostable variant of CRISPR-Cas9 system, CaldoCas9 from *Thermus thermophilus*, has been shown capable of genome editing at up to 65$^{\circ }$C [102], and CRISPR-Cas9 coupled with homology-directed repair has been used to integrate mutant alleles with a unique 24 nt ‘bookmark’ sequence into the *C. botulinum* genome, enabling precise gene replacement [103]. Stable integration has also been demonstrated in *Pseudonocardia alni* using the $\varphi $C31 integrase delivered by conjugation from *E. coli* [104].

For non-model fungi, *Agrobacterium tumefaciens*-mediated transformation (ATMT) remains the go to method for genomic integration with a binary vector that includes fungal-compatible promoters and homology arms for targeted integration. This has been shown to work effectively in *Aspergillus nidulans* strains [105]. Scaling the complexity of the integrated payloads, the ‘Simultaneous in vivo Assembly and Targeted genome Integration of Multiple DNA fragments’ (SATIMD) method has also been developed that enables *in vivo* DNA assembly and targeted genome integration through homologous recombination induced by CRISPR/Cas9-mediated cutting. This approach has been shown able to precisely integrate large constructs (up to 32.7 kb demonstrated) in a single transformation step [106].

For non-conventional yeasts, recent genome integration strategies have improved through the optimization of both homologous recombination and nonhomologous end joining (NHEJ) mediated CRISPR/Cas systems [107–109]. In *Yarrowia lipolytica*, a homology-independent CRISPR/Cas9-based system achieved one-step targeted genome integration without the need for homology arms or selection markers. This method achieved up to 55% integration efficiency and allowed iterative insertion of multiple genes, including an 8.4 kb biosynthetic fragment [107]. In addition, the Golden Gate-based NHEJ integration system YALIcloneNHEJ has been shown capable of multi-gene assembly and random genomic integration of large DNA constructs, with the *in vivo* assembly of up to 10 fragments [108], while the YaliCraft CRISPR/Cas9 toolkit has demonstrated improved precision through Cas9-mediated double-strand breaks and homology-directed repair, as well as the ability for rapid retargeting of multi-gene integration cassettes to alternative genomic loci [109].

Despite these advancements, endogenous CRISPR systems often negatively impact the function of genome integration tools. For example, active CRISPR interference frequently recognizes and cleaves heterologous DNA, significantly reducing transformation success and plasmid stability [110, 111]. Furthermore, in the anaerobe *Clostridium butyricum*, the introduction of a Cas9-expressing plasmid proved highly toxic, resulting in extremely poor recovery of transformants [112]. However, in some cases repurposing the host’s native CRISPR-Cas system has enabled efficient plasmid delivery as this approach helps avoid self-targeting interference [111, 113].

### Common challenges with existing methods

The genome integration tools discussed so far are mostly derived from naturally occurring systems evolved for roles such as phage replication, host immunity, and horizontal gene transfer. While powerful, their portability is often limited to specific hosts. For example, the lambda Red recombination system functions with high efficiency in *E. coli*, but performs poorly in distantly related microbes due to host-specific cofactors and misregulation of its components, especially for dsDNA recombination [114]. Similarly, CRISPR-based systems can cause toxicity or low expression in non-native hosts [115, 116], and the increasing size of payloads researchers wish to integrate often exceeds the natural limits of these systems.

Another issue is that integration efficiency is highly influenced by sequence and structural features of the host genome [117, 118]. For example, transposons insertion efficiency is typically higher towards the origin of replication and they are known to interact better with negatively supercoiled DNA rather than positively supercoiled or nicked DNA [119]. Another frequently observed bias in chromosomal transposons is their preference for inserting into target sites near their original location, in a phenomenon known as ‘local hopping’ [119]. Similar biases are also mirrored in phage integrase-mediated approaches that exhibit varying efficiency across different target sites. For example, in *E. coli* the phage 186 integrase has an efficiency $\sim $500-fold higher than Phi80, likely because it targets a site closer to the genome’s origin of replication, which has a more accessible chromatin structure. Phage integrase-mediated approaches are also constrained by predetermined integration sites, which limit the number and position of possible insertions into a genome. More generally, epigenetic modifications like DNA methylation can also impact the efficiency of transposases and integrases, both in detrimental and beneficial ways [120, 121]. To date, our understanding of the effects of epigenetic modification on genome integration tools is limited.

Genomic modifications themselves can also introduce long-term complications, particularly when multiple rounds of modification are required. For example, scars left by integrase and transposon mechanisms can accumulate and compromise genome stability, thereby limiting future modifications of the host. Several approaches have been developed to overcome this issue, such as counter-selection replacement via lambda Red for markerless chromosome integration [122], or the use of a dual selection marker called tetAOPT, which enables high efficiency (up to 50% in *E. coli*) without introducing scars [123]. However, these methods complicate the integration procedure and lead to extended experimental timelines.

Challenges also arise in developing tools that can be effectively used across diverse species. Differences in codon usage, transformation efficiency, regulatory parts, and endogenous restriction enzyme systems can all cause issues with the transfer of a tool from one organism to another. Many of these issues can be addressed. For example, codon usage can be harmonized for multi-kingdom applications [124, 125], endogenous restriction enzyme defence mechanisms can be evaded by *in vitro* methylation of DNA prior to transformations [126], and organizations like Cultivarium are creating standardized protocols for high efficiency transformation of diverse organisms [127], as well as generating libraries of regulatory parts compatible across multiple species [128]. Though difficult, systematically addressing each of these hurdles in a single pipeline is potentially feasible, especially given recent advancements in enzyme-based *de novo* DNA synthesis technologies [129–131], which remove the barrier to constructing large and bespoke genome engineering systems. These new DNA synthesis technologies will be particularly useful when working with eukaryotes, where extensive refactoring of systems can be necessary [132].

### Emerging opportunities via synthetic genomics

An exciting route to overcome these challenges is to engineer genomes from the ground up with features that simplify the later insertion of additional genetic material. The emerging field of synthetic genomics [133] is beginning to make this vision a reality, having demonstrated the ability to synthesize recoded genomes of bacteria [134] and eukaryotic cells [135] with new functionalities (e.g. the ability to perform accelerated evolution using the SCRaMbLE system [135]).

The simplest genome-wide modification to facilitate later modifications would be the strategic introduction of numerous integrase sites at genomic locations that do not interfere with endogenous functions. This could be achieved by harnessing the growing library of well-characterized recombinases and their unique attB sites [136], or through the incorporation of efficient CRISPR/Cas binding sequences with corresponding guide RNAs (gRNAs) [6].

Synthetic genomics could also support the creation of organisms purposefully designed with enhanced integration capabilities, eliminating the need for external delivery of these tools. This could involve constitutive or inducible expression of tightly regulated recombinases, optimized DNA repair pathways, or entirely novel recombination systems engineered for high-efficiency integration. The genome architecture itself could also be redesigned to include dedicated ‘integration zones’ reserved for foreign DNA, which are insulated from the broader genomic context (e.g. local transcription and strong chromatin structure). Such modifications would dramatically simplify strain engineering and mirror the expanding use of cell-free systems as ‘breadboards’ for testing synthetic pathways, allowing genome designs to be rapidly prototyped and iterated upon [137].

The idea of creating organisms with genomes specifically designed for extensive modification after their construction would also open new possibilities for crafting engineered cells that can be rapidly repurposed over time for diverse and changing applications. Longer term, this approach could even enable the development of strains with the capability to modify their own genomes in response to environmental signals or programmed instructions—essentially engineering cells capable of evolving in new but partially defined ways [138–140]. Such self-modifying biological systems could dynamically adapt to changing production requirements, optimize metabolic output, or evolve new traits, transforming how we think about biological design and its link with evolution [141].

Such an approach has in part begun to be explored in the context of the multi-stage SCRaMbLE-in methodology for optimizing metabolic pathways in *Saccharomyces cerevisiae* [142]. SCRaMbLE-in starts by performing a constrained *in vitro* recombination reaction to diversity gene expression of a target pathway in a controlled manner (i.e. only affecting the regulation of certain genes). Then, these diverse pathways are integrated into *S. cerevisiae* cells containing synthetic yeast chromosomes harbouring LoxPsym sites, and their wider genome SCRaMbLE’ed before artificial selection is applied to extract promising candidates—effectively evolving the population towards a desired characteristic.

## Discussion

As genome engineering technologies continue to evolve, they are paving the way for more precise and comprehensive modification of diverse bacterial species. The convergence of CRISPR-based systems, recombinase technologies, and synthetic genomics promises to address many current limitations while also opening up entirely new possibilities for biological design.

The cross-kingdom transfer of technologies represents an exciting prospect for yet further expansion of the bacterial genome engineering toolkit. Recent mammalian genome integration tools such as PASTE [143] combine CRISPR-Cas9 nickases with reverse transcriptases and serine integrases to enable insertions of up to 36 kb DNA payloads without double-strand breaks, while PASSIGE [144] employs continuously evolved Bxb1 recombinase variants (evoBxb1 and eeBxb1) that demonstrate 16-fold higher efficiency than PASTE. Adapting these mammalian systems for use in bacterial hosts presents both opportunities and challenges. While serine integrases and CRISPR systems are known to function within bacteria, successful implementation will require addressing bacterial-specific constraints including the expression and proper folding of large fusion proteins like Cas9-RT, the availability of compatible attB sites, and tuning genetic regulation to ensure high editing efficiency.

Advances in high-throughput assembly methods [145] and direct DNA synthesis using engineered polymerases [130, 131] are dramatically reducing the time and cost associated with constructing large genetic payloads and increasing the scale at which genome integration technologies must perform. In addition, artificial intelligence (AI) and machine learning are emerging as powerful aids for genome engineering [146–148]. AI-driven tools are increasingly capable of predicting optimal integration sites, designing gRNAs with minimal off-target effects [149], and optimizing genetic circuit architectures for specific chromosomal contexts [150, 151], with many of these areas already seeing the release of publicly available tools that have helped increase adoption. Automation efforts are also likely to be crucial for scaling genome engineering efforts from research proofs-of-concept to more substantial industrially relevant applications, with the development of standardized parts, protocols, and quality metrics helping to support more reproducible and predictable genome engineering outcomes [152].

Beyond the development of genome engineering methods, it is clear that supporting analysis tools will be required to verify and characterize the increasingly substantial modifications that are made. Emerging sequencing technologies such as POLAR-seq [153] enable comprehensive validation of chromosomal modifications, while targeted long-read sequencing [154, 155] would allow for detailed and cost-effective analysis of integration events across diverse genetic design pools. The further integration of multi-omics analysis of engineered cells (e.g. combining RNA sequencing and ribosome profiling [156, 157]) would provide deeper insight into the regulatory effects that genome modifications have, increasing our ability to ensure new biological functions are achieved as intended. Although these methods are not widely employed today, as the scale of genome modification grows, it is likely their use will become essential for allowing informed genetic design decisions that support the rapid optimization of desired functionalities [158].

In parallel, it is crucial that regulatory and safety frameworks evolve alongside these emerging technological capabilities [159–161]. As genome integration tools become more powerful and accessible, robust containment strategies, risk assessments, and regulatory guidelines will become necessary for responsible development and deployment. This may include the development of reversible integration systems that can rapidly remove inserted genetic materials that fail to function as expected, the implementation of multilayered containment strategies to safeguard against unintended modifications, and the establishment of clear protocols for reproducibility [162].

The speed at which advances are being made suggests that we will soon reach an era of virtually unconstrained bacterial genome modification. At that point, our primary limitations will shift from technical to conceptual and societal, centred around our understanding of how biology is best engineered and the appetite of society to harness the diverse and powerful capabilities of biology to tackle global challenges.

## Materials and Methods

### Citation analysis

Data for the citation analysis (Fig. 1; Supplementary Data 1) was performed using Google Scholar, searching for all articles by year citing the main references for each genome editing methodology: Lambda Red chromosomal knock-outs [9]; Lambda Red protocol [28]; INTEGRATE [17]; Transposon-encoded CRISPR-Cas [6]; Synthetic Landing Pads [4]; pIT5 Plasmid Series [53]; One-Step Cloning [22]; CRIM [8].

## Supplementary Material

data-s1_ysaf019

## Data Availability

Data for the bibliographic analysis presented in Fig. 1 are available as Supplementary Data 1.
